# Case Report: *Leishmania* and HIV Co-Diagnosis: How to Understand Medical History?

**DOI:** 10.3389/fimmu.2021.669723

**Published:** 2021-04-21

**Authors:** Arthur Dujardin, Arnaud de La Blanchardière, Julia Dina, Karl Stefic, Christophe Ravel, Julie Bonhomme, Renaud Verdon, Anna Lucie Fournier

**Affiliations:** ^1^ Infectious Diseases Department, Caen University Hospital, Caen, France; ^2^ Virology Department, Normandie Univ, UNICAEN, Normandie University Hospital, Caen, France; ^3^ Normandie University, UNICAEN, Groupe de Recherche sur l’Adaptation Microbienne (GRAM 2.0), Normandie Univ, UNICAEN, UNIROUEN, GRAM 2.0, Caen, France; ^4^ Virology Laboratory, Tours Hospital, Tours, France; ^5^ National Reference Centre for Leishmaniasis, University Hospital Centre of Montpellier, MiVEGEC, University of Montpellier, Montpellier, France; ^6^ Normandie University, UNICAEN, U2RM EA2128, Microbiology Department, Normandie University Hospital, PFRS, Caen, France

**Keywords:** visceral leishmaniasis, HIV, hemophagocytic lymphohistiocytosis, bone marrow aspirate, pancytopenia

## Abstract

We report a case of a severe visceral leishmaniasis revealing an HIV-1 infection presenting as an acute primary infection. A young French man living in Paris with history of unprotected sex with a recent male partner and recent travel in Greece was admitted in our Infectious Diseases Department, presenting with acute febrile psychotic disorder, and positive HIV-1 serology with high viral load, very low CD4^+^ T-cells count and a western blot pattern suggesting an acute infection. The psychotic disorder was finally related to hemophagocytic lymphohistiocytosis diagnosed on bone marrow aspiration, supposedly secondary to HIV acute primary infection. The progressive worsening of pancytopenia despite antiretroviral treatment and the persistence of fever, chills and sweat led to the diagnosis of visceral leishmaniasis through bone marrow biopsy and leishmanial serology. He was treated with intravenous liposomal amphotericin B with quick improvement. We discuss the way HIV infection and visceral leishmaniasis may have interact to lead to the clinical presentation of our patient.

## Background

In 2019, 1.7 million people were newly infected by HIV and 690 000 people died from AIDS-related illness worldwide (1). Acute HIV infection can regroup various non-specific symptoms such as fever, maculopapular rash, pharyngitis, enlarged lymph nodes or diarrhea and occur two to six weeks after contamination. Viral load is at its highest point during early infection along with inflammatory state and CD4^+^ T-cell depletion. Then, without care, after a transient recovery, CD4^+^ T-cells count slowly decreases to reach chronic infection and AIDS. Identifying persons at the very early stage of infection or within weeks of HIV antibody seroconversion is important for both clinical decision and prevention but may be challenging. Acute infection diagnosis is based on the presence of p24 antigen and/or HIV RNA in serum or plasma in the absence of detectable HIV antibodies. A recent infection can be suspected if the immunoblot displays a negative or incomplete pattern. During the late stages of the course of HIV infection the specific antibodies are lost and can lead to a weakly reactive immunoblot (2).

Leishmaniasis is a sandfly-borne protozoan parasitic disease. Visceral leishmaniasis (VL) is the disseminated form of *Leishmania donovani* or *infantum* infection with potential severe complications (cachexia, hepatic dysfunction, hemorrhages and - almost always without proper treatment - death). Leishmaniasis is considered as one of the 13 “core” neglected tropical diseases in the world with 50 000 to 90 000 estimated cases of VL each year worldwide (3, 4). In 2015, 90% of new VL cases reported to WHO occurred in 7 countries (Brazil, India, Ethiopia, Kenya, Somalia, South Sudan and Sudan) (5).

VL is associated with HIV infection in the Mediterranean area and behaves as a major opportunistic infection although not considered an AIDS classifying event (6). HIV and leishmaniasis co-infection associated physiopathology was described by Alvar et al. Clinical presentation of visceral leishmaniasis in immunocompromised hosts can be atypical (7). Control of leishmaniasis relies upon a Th1 CD4^+^-based immune response, which is deficient during HIV infection, leading to easier dissemination of the disease. When HIV infection is unknown or treated, quiescent *Leishmania* can uncover itself because immunity is affected by HIV. Reciprocally, *Leishmania* infection promotes HIV progression and CD4^+^ T-cells counts are significantly lower in HIV infected patients suffering from co-infection (6). Introduction of combined antiretroviral therapy (cART) was followed by a decreased incidence of VL in Southern Europe (8).

We describe in this case report a *Leishmania* and HIV-1 co-infection with atypical presentation.

## Case Description

A 25-year-old man with no medical history living in Paris, France, went to the local emergency room in November 2018 with sudden onset of psychotic symptoms (paranoid delusions, auditory hallucinations and major anxiety) associated with fever, cough and general asthenia. He had no psychiatric history apart from an anxious personality and a mild tobacco and cannabis consumption and was not taking any medication. He had travelled in Greece for one week and had returned ten days before the first symptoms. He reported having unprotected sex with a man 2 months before and during his travel.

On admission, he had fever (38.5°C), pharyngitis, oral candidiasis and skin rash. Laboratory values revealed increased C-reactive protein (18 mg/L), mild anemia (10.5 g/dL) with low reticulocytes count (37 G/L), lymphopenia (500/mm^3^), neutropenia down to 600/mm^3^ and thrombocytopenia (90 G/L). HIV-1 serology returned positive, confirmed by immunoblot, with a viral load of 5.47 million copies/mL and a CD4^+^ T-cells count of 90/mm^3^. Neuroleptic drugs (cyamemazine, risperidone) and benzodiazepines were introduced to treat psychotic and anxious symptoms. He was transferred to our Infectious Diseases Department. HIV blood viral load at admission (2 weeks after first symptoms) was at 6.85 million copies/mL with a blood CD4^+^ T-cells count of 30/mm^3^ and a global lymphopenia (including CD8^+^ and NK cells). The immunoblot showed an uncommon band pattern (gp120 and gp41 reactivity, weak ambiguous reactivity to p24 and p31, no reactivity to other antigens), which could have been consistent with acute primary infection. He never had any HIV infection screening in the past. The research of past blood tests showed a normal lymphocytes count (1.4 G/L) a year ago. Of note, his recent partner was just diagnosed with HIV infection as well.

Lumbar puncture, electroencephalography and cerebral MRI showed no abnormality. Thoraco-abdominal and pelvic CT revealed homogenous hepatosplenomegaly and few parenchymatous interstitial pulmonary lesions (micronodules and reticulations). Sputum cultures and bronchoalveolar lavage showed no *Pneumocystis* nor tuberculosis infection. No co-infection with HAV, HBV, HCV, HEV, toxoplasmosis nor syphilis was found. EBV and CMV viral loads were undetectable. Genotypic testing found a CRF19-cpx strain. Combined anti-retroviral therapy (cART) was introduced 2 weeks after diagnosis, consisting in tenofovir, emtricitabine and raltegravir, and *Pneumocystis* pneumonia was prevented by cotrimoxazole.

Despite cART and neuroleptic drugs, psychotic symptoms and fever persisted associated with chills, sweats and a neurological disorder occurred with bilateral muscle rigidity. The worsening of his clinical state required intensive care. A neuroleptic malignant syndrome was diagnosed, and extrapyramidal syndrome recovered after stopping neuroleptic treatment, but fever, chills and night sweats persisted. At this time, blood tests showed worsening pancytopenia (anemia 7.0 g/dL, platelets count 50 G/L, neutropenia 600/mm^3^), hyperferritinemia, elevated liver enzymes and mild elevated triglyceridemia. We hypothesized that the patient had a hemophagocytic lymphohistiocytosis (HLH) triggered by an acute primary HIV infection. Sternal bone marrow aspiration was performed showing signs of macrophages activation without hemophagocytosis. No *Leishmania* was found on bone marrow aspirate smears and *Mycobacterium tuberculosis* complex PCR was negative. An empiric treatment with intravenous immunoglobulins 1 g/kg for 2 days was administered; no clinical response was observed.

**Figure 1:**
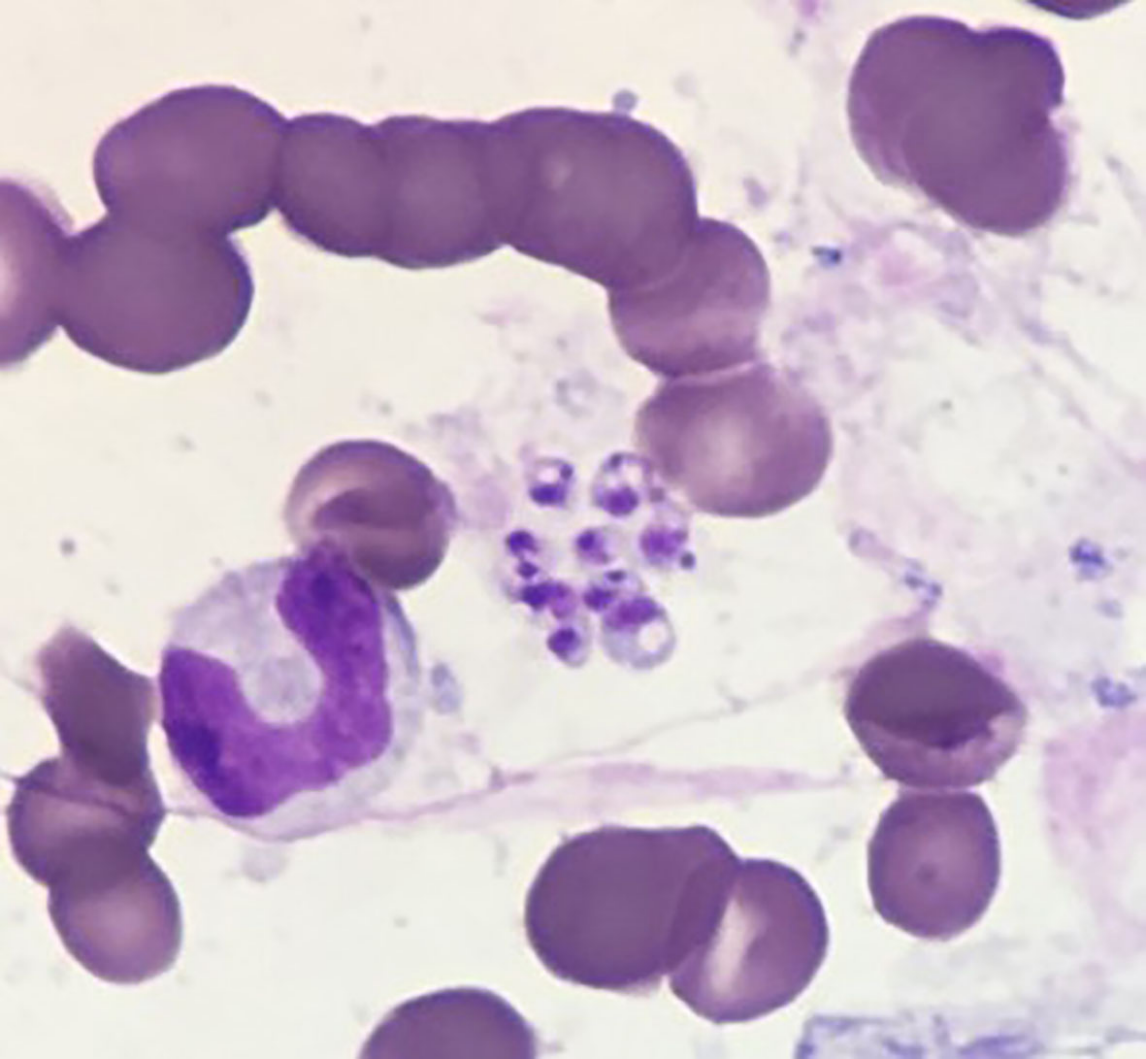
Bone marrow aspirate smear (from bone marrow biopsy) stained with May-Grünwald Giemsa showing amastigote forms of *Leishmania* spp.

Finally, a bone marrow biopsy was performed showing hemophagocytosis, confirming HLH, and moreover, revealing the presence of *Leishmania* spp (**Figure 1**), with a low parasitic load. Serology was also positive for *Leishmania infantum* antibodies by immune-enzymatic screening technique, confirmed by Western-Blot assay. Molecular species identification confirmed a *Leishmania infantum* strand.

**Figure 2:**
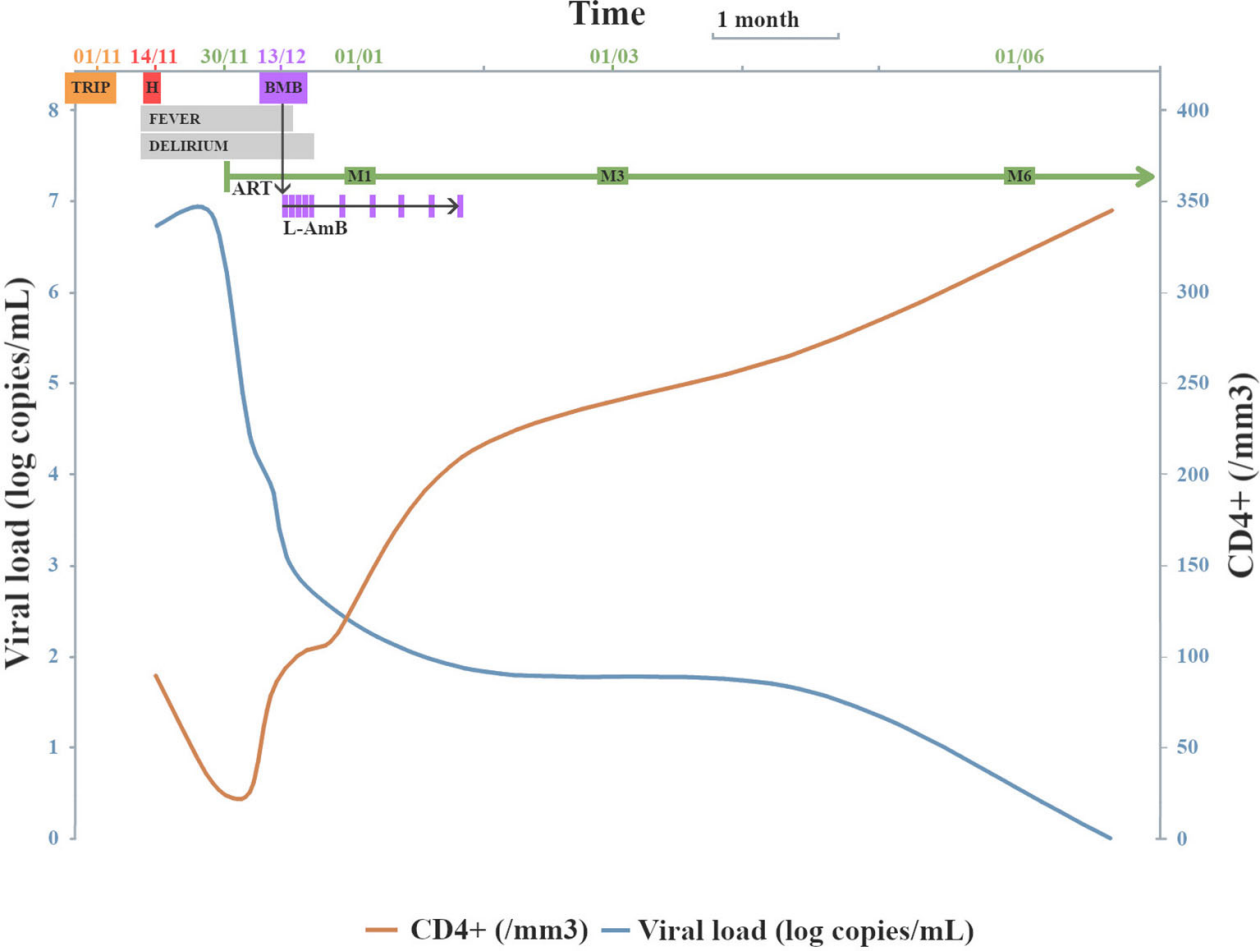
Viral load and CD4^+^ T-cells count evolution following cART initiation and VL diagnosis. TRIP = travel in Greece; H = date of hospitalization; (c)ART = (combined) anti-retroviral therapy; BMB = bone marrow biopsy (VL diagnosis); M1 = first month after cART initiation; M3 = third month after cART initiation; M6 = sixth month after cART initiation; LAmB = intravenous liposomal amphotericin B administrations.

Intravenous liposomal amphotericin B was administered at 4 mg/kg/day on five consecutive days followed by five weekly administrations, allowing complete clinical recovery and pancytopenia recovery. Patient was discharged, no secondary prophylaxis against VL was prescribed. Viral load decreased to 75 copies/mL and CD4^+^ T-cells count increased to 213/mm^3^ two months after cART initiation and viral load was undetectable with CD4^+^ T-cells count at 345/mm^3^ after 6 months (**Figure 2**), with no sign of VL relapse. After 24 months of follow-up, no VL relapse was observed.

Thus, the diagnosis of secondary HLH caused by visceral leishmaniasis and HIV co-infection was established. In order to date the HIV infection, an incidence immunoassay designed for HIV surveillance and based on antibody binding to the gp41 and gp120 glycoproteins was performed retrospectively on the two samples with positive immunoblot, which had identical patterns. Results of this test were not in favor of an infection less than six months old. This case needed discussion within a multi-disciplinary staff with infectious diseases clinician, virologist and parasitologist but we couldn’t conclude regarding the stage of the HIV infection.

## Discussion

We present a case of a *L. infantum* and HIV co-infection in a patient returning from Greece, who presented with psychotic signs, fever, and pancytopenia leading to the diagnosis of HLH due to visceral leishmaniasis. The clinical and biological features of HIV infection were consistent with an acute primary infection, but incidence immunoassay was not. HLH, which was believed to be due to HIV, turned out to be due to visceral leishmaniasis. Thus, the diagnosis was done after overcoming difficulties that we believe interesting to discuss hereafter.

On one hand, the patient’s recent history of unprotected sexual intercourse, his normal lymphocytes count one year before hospitalization, his new partner just being diagnosed with HIV infection as well, the high viral load at the time of diagnosis, the incomplete band pattern of the immunoblot and the quick increase in CD4^+^ T-cells count are all consistent with an acute primary HIV infection. On the other hand, the very low CD4^+^ T-cells count, the identical pattern of the immunoblot during six weeks apart, and the results of the incidence immunoassay for recent infection are in favor of an infection older than six months (9). The viral strain CRF 19-cpx was reported as a virulent strain with a rapid progression towards immunosuppression (10). This could explain the conflicting results of this clinical presentation and of immunological and virological markers. The immunosuppression status with low CD4^+^ T-cells count can result in a loss of antibodies, an incomplete western blot and can even lead to false recent results of so-called incidence immunoassays, which affects their specificity (11).The very low CD4^+^ T-cells count observed in our patient could have been explained by the global lymphopenia due to the HIV infection and the associated VL. Indeed, patients co-infected with VL and HIV have lower CD4^+^ T-cells count than HIV-infected patients only (8). VL is responsible for an inflammatory state that could be caused either by immune activation in response to quiescent amastigote intracellular parasites and/or by intestinal microbial translocation linked to mucosal invasion by amastigote forms (12, 13). Furthermore, in infected cells, leishmaniasis is responsible for an overexpression of CCR5, a coreceptor necessary to HIV entry into T-cells, promoting increased viral load and disease progression towards AIDS (14). We know that opportunistic infections can occur during the early stages of HIV infection as *Pneumocystis* pneumonia, *Candida* esophagitis, toxoplasmosis or cryptococcal meningitis have been described during primary HIV infection (15).

VL infection could have been promoted by HIV co-infection. Indeed, VL is challenged by a CD4^+^ based Th1 immune response, increasing IFN-γ production which contributes to defense against intracellular bacterias, virus and parasites. However, HIV infection alters T-cells responsiveness to *Leishmania* and is associated with low levels of IFN-γ. Moreover, because of a predominant Th2 response with high levels of IL-10, the immune response against protozoans such as *Leishmania* is poorly effective (16). Furthermore, it has been shown that HIV-infected patients had low levels of IL-15 whereas patients presenting with VL and a clinical response to specific treatment had increased levels of IL-15 suggesting its role in VL control through CD8^+^ cytotoxic T-cells activation (17). HIV infection is a risk factor of VL primary infection and reactivation (16). In this case report, we didn’t observe a link between parasitic load and the low level of CD4^+^ T-cells count but the negative first bone marrow examination and the low parasitic load are in favor of a primary *Leishmania* infection. In immunocompetent hosts, parasitological demonstration is the gold standard for VL diagnosis. Microscopic visualization of amastigotes can be achieved using splenic, bone marrow or lymph node aspirates with sensitivity of 93 to 99%, 53-86% and 52-58% respectively (18). VL diagnosis in immunocompromised hosts usually need repeated samples of multiple origins (bone marrow aspirate, peripheral blood PCR, serology, liver, digestive tract or lower airways biopsy) (8). In this case, the patient’s life was endangered and whereas the first bone marrow aspirate by sternal puncture yielded no diagnosis, iliac bone marrow biopsy fortunately led to the diagnosis and appropriate treatment.

This case is a rare report of acute psychotic disorder without meningitis or encephalitis in the setting of VL-HIV coinfection and HLH. Atypical clinical presentations of VL in immunocompromised hosts have been reported with peripheral and central nervous system disorders (8, 19), but psychiatric disorders were very rarely described (20, 21). To our knowledge, no case of VL-HIV coinfection with psychiatric disorder at presentation were published in the medical literature. On the other hand, HLH can, unusually, present with psychiatric changes (22, 23). Regarding the clinical and biological presentation of our patient, we discussed the diagnosis of an HIV-associated immune reconstitution inflammatory syndrome (IRIS). VL-related IRIS is uncommon, 19 cases were reported in a systematic literature review. Mean time between ART initiation and VL-related IRIS symptoms was 4 months (range: 6 days – 111 months) (24). We did not retain this hypothesis because our patient systemic inflammation response and HLH signs occurred before ART initiation.

HLH is a life-threatening condition associated with pancytopenia, persistent fever and hepatosplenomegaly. Its physiopathology relies on two conditions: natural killers and cytotoxic T-cells deficiency and macrophagic activation leading to a cytokine storm causing unchecked inflammation. Indeed, secondary HLH generally occurs in patients with an underlying predisposition such as hematologic malignancies or autoimmune diseases and triggered mostly by infections. HIV infection can act as a predisposing immunocompromising disease and as a trigger itself. A meta-analysis conducted by Ramos-Casals et al. referenced 173/2197 (8%) HIV-associated HLH cases between 1974 and 2011 (25). More recently, a systematic review of HIV-associated HLH found only 8/52 (15%) patients with no trigger identified highlighting the predisposing role of HIV but its scarce implication as a trigger of HLH (26). This suggests the importance to look for another cause than HIV itself in case of HIV-associated HLH. Moreover, etiologic treatment is the best way to cure the patient, therefore etiologic diagnosis must be rapidly obtained.

This uncommon case is an example of HIV and VL dangerous co-infection worsened by HLH. It highlights the need to track down VL when HIV infected patients present with fever and/or pancytopenia without improvement after cART initiation. In immunocompromised hosts, it is important to remember that biological and histological samples must be repeated.

## Data Availability Statement

The original contributions presented in the study are included in the article/supplementary material. Further inquiries can be directed to the corresponding author.

## Ethics Statement

Written informed consent was obtained from the individual(s) for the publication of any potentially identifiable images or data included in this article.

## Author Contributions

AD and AF wrote the first draft and the manuscript. RV, AB, JD, KS, CR and JB reviewed the draft and the manuscript. All authors contributed to the article and approved the submitted version.

## Conflict of Interest

The authors declare that the research was conducted in the absence of any commercial or financial relationships that could be construed as a potential conflict of interest.
